# Subnanometer imaging and controlled dynamical patterning of thermocapillary driven deformation of thin liquid films

**DOI:** 10.1038/s41377-019-0190-6

**Published:** 2019-08-28

**Authors:** Shimon Rubin, Brandon Hong, Yeshaiahu Fainman

**Affiliations:** Department of Electrical and Computer Engineering, University of California, San Diego, 9500 Gilman Dr., La Jolla, CA 92023 USA

**Keywords:** Optical physics, Nanophotonics and plasmonics

## Abstract

Exploring and controlling the physical factors that determine the topography of thin liquid dielectric films are of interest in manifold fields of research in physics, applied mathematics, and engineering and have been a key aspect of many technological advancements. Visualization of thin liquid dielectric film topography and local thickness measurements are essential tools for characterizing and interpreting the underlying processes. However, achieving high sensitivity with respect to subnanometric changes in thickness via standard optical methods is challenging. We propose a combined imaging and optical patterning projection platform that is capable of optically inducing dynamical flows in thin liquid dielectric films and plasmonically resolving the resulting changes in topography and thickness. In particular, we employ the thermocapillary effect in fluids as a novel heat-based method to tune plasmonic resonances and visualize dynamical processes in thin liquid dielectric films. The presented results indicate that light-induced thermocapillary flows can form and translate droplets and create indentation patterns on demand in thin liquid dielectric films of subwavelength thickness and that plasmonic microscopy can image these fluid dynamical processes with a subnanometer sensitivity along the vertical direction.

## Sensitive mapping of liquid dielectric topography and thickness variation

A technique that induces and maps tiny topographical changes in thin liquid dielectric films could have applications in interfacial science and industry. Shimon Rubin, Brandon Hong and Yeshaiahu Fainman of the University of California, San Diego used a heating laser to induce thermocapillary flows in thin liquid dielectric films. These films are used, among various things, for UV photolithography of semiconductors and microelectronics applications. The team mapped the changes caused by the flows using surface plasmon resonance microscopy, a non-contact imaging method that measures changes of the collective oscillations of surface electromagnetic waves and electrons in the metal substrate. The technique successfully identified subnanometer-scale topographical changes caused by the thermocapillary flow. It overcomes problems in currently used techniques, such as white light interferometry, which is not sensitive enough to detect small, local thickness variations. The technique has many potential applications, including the study of light-induced thickness changes in photosensitive materials.

## Introduction

Determining the topography of thin liquid dielectric (TLD) films is of fundamental importance for the basic studies of interfacial science, such as the study of wettability and spreading^1^, the study of the response to external stimuli in both physical^2^ and biological systems^3^, and studies related to numerous industrial applications, such as coatings, insulating layers and surface modifiers^4,5^. While one of the commonly used methods to determine the thickness of TLD films is white-light interferometry, the sensitivity of this method to local small thickness variations is low, especially when the film thickness is less than the optical wavelength due to the low reflection from the film’s surface. One attractive possibility to enhance this sensitivity is to utilize strong coupling between collective oscillations of electrons in the metal (surface plasmons) to the resulting radiated electromagnetic field (the polariton), which supports surface plasmon polaritons (SPPs) - electromagnetic waves that propagate along the metal-dielectric interface and strongly decay in the direction perpendicular to it^6^. This localization property makes SPPs very sensitive to the dielectric properties of materials in close proximity to the metal surface, which, over the last few decades, has led to a wide spectrum of sensing and imaging techniques, such as surface plasmon spectroscopy and surface-plasmon resonance microscopy (SPRM)^7^. In particular, SPRM is a well-established method for label-free imaging of low contrast features such as the roughness of thin solid dielectric films^8^, imaging of small biological objects such as bacteria, viruses, and biofilms, and the detection of analytes as a result of surface reactions. The latter typically results in a few-nanometer-thick monolayer array of molecules bound to metal surfaces, which modifies the SPP momentum (e.g., by changing the refractive index), and numerous applications, such as the biosensing of surface reaction events^9,10^ and cell adhesion sites^11^, have been found. Unlike alternative methods suited to characterizing nanometric and subnanometric topographies, such as atomic force microscopy (AFM), which are scanning imaging techniques, SPRM is an optical technique that can flexibly measure topographies in both wide-field imaging and scanning confocal modes. Moreover, SPRM is a noncontact method that avoids the sample-specific mechanical interactions involved in AFM imaging. In particular, for fluid topometry (i.e., topography measurement), noncontact optical imaging avoids sensitive perturbations to the TLD dynamics that would otherwise be inevitable with contact mechanical probing.

In this work, we demonstrate for the first time a combined imaging and optical patterning projection platform that is capable of optically inducing dynamical processes in TLD films and then measure subnanometric topographic and thickness changes. Specifically, the optically projected pattern induces thermal gradients that in turn trigger thermocapillary (TC) flows, while SPRM imaging leverages the near-field profile of surface plasmon resonances to resolve and characterize the subnanometric topographies of flow-induced interface perturbations. Analogous to traditional pump-probe systems, our system uses a heating pump beam to invoke TC flows and is capable of probing the accompanying deformation of the TLD film.

In our work, we introduce local changes in TLD film thickness by taking advantage of the characteristic optical absorption in metals (i.e., unrelated to SPP-driven heating), which stimulates the TC effect^12^. The latter is a special case of the Marangoni effect, which is manifested by TC flows and the deformation of a TLD film due to the spatially non-uniform temperature distribution of the liquid-gas interface and subsequent spatial variations of the surface tension. Notably, in previous works, heat-induced tuning of the SPP coupling condition was mostly invoked by thermo-optic and phase transition effects (see ref. ^13^ and references therein); in our work, heat-generated SPP tuning is experimentally triggered for the first time by a fundamentally different mechanism that stems from geometrical changes of the TLD film topography, as was recently theoretically demonstrated^14^.

Previous studies that applied SPRM imaging to thin dielectric films, such as^15^, focused on solid and static films and did not employ the SPRM method to study fluid dynamical processes. Furthermore, while TC-induced patterning has received significant attention in the past, mostly in the context of thin polymer film molding, where TC flows are triggered only in regions where the polymer film is above the glass phase transition temperature^16^ (and also ref. ^17^ and references therein), we here leverage the ability to induce optically desired heating patterns to demonstrate the formation of light-induced droplets of different size directly from TLD film and their translation along the substrate (see also ref. ^18^ and ref. ^19^, which report the formation of droplets from amorphous silicon and gold by employing phase plates). To the best of our knowledge, previous studies in micro- and nanofluidics reported controlled droplet formation in microchannels by employing directional flows of several liquid phases (see ref. ^20^ for a recent review and references therein) and by utilizing a light-driven TC effect;^21^ however, these studies did not employ optical patterns to take advantage of the TC effect for the formation and translation of microdroplets directly from stationary TLD films without microchannels.

We experimentally employ traditional SPRM to image the dynamical depth changes and 3D nanoscale topography of the TLD film in a subwavelength thickness regime, which is challenging for purely interferometric methods. A numerical model is constructed that relates TLD thickness to the measured SPP coupling angle, allowing for the experimental determination of the TLD topography and local thickness from a series of SPRM frames. While in our work we experimentally determined the topography of TLD films with a mean thickness comparable to or smaller than the penetration depth of an SPP mode in the direction normal to the metal surface, we theoretically note that, in principle, it is possible to achieve similar sensitivity for subnanometer thickness changes of significantly thicker TLD films by leveraging waveguide (WG) modes, which admit the highest optical intensity within the film.

**Fig. 1:**
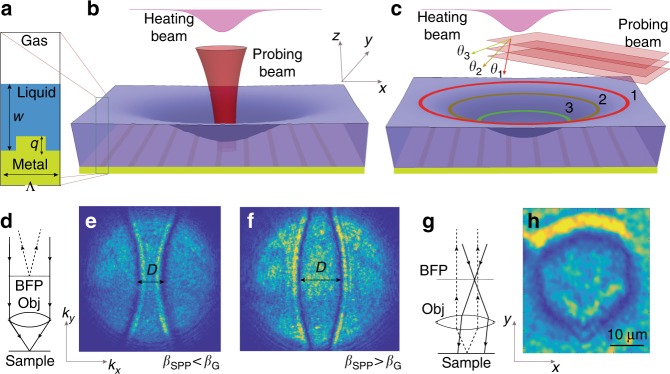
Schematic description of the working principles behind the TLD film topography measurement. **a** The system under study; a metal grating of period Λ and depth *q* covered with a TLD film of local thickness *w*. **b** *k*-space method: the probing focused Gaussian beam illuminates a small (diffraction limited) spatial region. **c** Real-space method: the probing beam is a plane wave of fixed directionality that covers a wide field of view of several tens of microns. **d**, **g** The corresponding schemes highlighting the key differences between the topometry methods. **e**, **f** Experimental representative images of the BFP with dark arcs indicative of the resonant coupling angles to SPP for the regimes *β*_*SPP*_ < *β*_*G*_ (**e**) and *β*_*SPP*_ > *β*_*G*_ (**f**), respectively. **h** Experimental representative image of a resonant contour due to an incident beam at some angle, which corresponds to a unique film thickness. By sweeping the angle of incidence (*θ*_1,2,3_ in (**c**)) of a given light source, the coupling to the SPP takes place at corresponding heights (see contours 1,2,3 in (**c**))

To illustrate the optical interaction with the TLD film for both manipulation and imaging, Fig. 1a presents key elements of our system under study: a metal grating of period Λ and wave vector *β*_*G*_ = 2*π*/Λ and an adjacent layer of the TLD film of thickness *w*, as measured from the bottom part of the grating grooves. Our imaging methods rely on capturing the angular and spatial content of the beam reflected from the sample, referred to below as *k*-space and real-space imaging, which correspond to the monochromatic coupling illumination configurations described in Fig. 1b, d and Fig. 1c, g, respectively. *k*-space imaging utilizes a probing focused Gaussian beam, produced by a collimated beam filling the objective back focal plane (BFP), which corresponds to a focused beam on the sample (Fig. 1b, d). Real-space imaging, on the other hand, utilizes the dual configuration with a plane wave made incident at a specified angle on the sample (Fig. 1c), which corresponds to a focused beam at the BFP (Fig. 1g). Both methods take advantage of the fact that the magnitude of the corresponding SPP momentum, *β*_*SPP*_, is a strictly increasing function of the TLD film thickness, which allows the construction of a one-to-one correspondence between the film thickness and the SPP resonant coupling angle.

Figure 1e, f presents representative *k*-space imaging results of the BFP, with inward and outward facing dark arcs separated by a distance *D* along the central line, which correspond to different SPP coupling regimes depending on the sign of the difference *β*_*SPP*_ − *β*_*G*_. Importantly, the distance *D*, which in our work can depend on time, together with the inward or outward pointing configuration of the dark arcs, uniquely sets the SPP coupling angle and the corresponding TLD film thickness at the focal point (see ref. ^22^ for BFP imaging due to reflectance from a metal grating covered with a solid dielectric film). The emergent patterns are related to the Kikuchi patterns or Kossel lines that have been observed in atomic structures through electron microscopy and Brillouin zone spectroscopy of photonic crystals^23^. In the real-space imaging setup (Fig. 1c, f), the regions of low reflected light are indicative of spatial regions where the resonant coupling condition of the incident plane wave into the SPP mode holds. Figure 1h presents a representative image of a wide field of view of a few tens of microns showing a dark contour indicative of fluid thickness that supports the SPP coupling condition at the illumination angle. By sweeping the incidence angle with the same source wavelength, one can then obtain a set of resonant curves that correspond to a set of level thickness contours where the incident plane wave couples to the SPP, which is schematically shown in Fig. 1c.

### Thin liquid film deformation due to optically induced thermocapillary effect

The topography of a TLD film is determined by the internal stress balance between capillary and viscous forces, as well as by external stimuli. Assuming a Newtonian fluid of viscosity *μ* (which holds for the silicone oil employed in our work), considering a linear dependence of the surface tension, *σ*, on the temperature difference Δ*T*^12^ given by

1 $$\sigma (T) = \sigma _0 - \sigma _T{\mathrm{\Delta }}T;\quad {\mathrm{\Delta }}T \equiv T - T_0$$

and applying the lubrication approximation for the corresponding linearized Navier-Stokes equation^2^ yields the following equation for the TLD film deformation *η* under spatiotemporal-dependent optical intensity *I*,

2 $$\frac{{\partial \eta }}{{\partial t}} + \nabla _\parallel ^4\eta = - {\mathrm{Ma}} \cdot \chi \cdot \frac{{\tau _l}}{{\tau _{th}}}I/2$$

(see ref. ^14^ and Supplementary Material). Here, *σ*_*T*_ is the so-called Marangoni constant; ${\mathrm{Ma}} \equiv \sigma _T{\mathrm{\Delta }}Tw_0/(\mu D_{th}^m)$ is the dimensionless Marangoni number, which represents the ratio between the surface tension stresses due to the TC effect and dissipative forces due to fluid viscosity and thermal diffusivity in a film of height *w*_0_; $\chi \equiv (\alpha _{th}^md^2I_0)/(k_{th}^m{\mathrm{\Delta }}T)$ is the dimensionless intensity of the heat source; *d* is the typical length scale along the in-plane direction; *I*_0_ is the characteristic optical power scale; $D_{th}^m \equiv k_{th}^m/(\rho ^mc_p^m)$ is the heat diffusion coefficient; *ρ*^*m*^, $c_p^m$, $k_{th}^m$, and $\alpha _{th}^m$ are the mass density, specific heat, heat conductance, and optical absorption coefficient of the metal substrate, respectively; $\tau _{th} \equiv d^2/D_{th}^m$ is the characteristic heat diffusion time scale in the metal; $\tau _l \equiv d^4/D_\sigma$ is the typical thin film deformation time scale; and $D_\sigma \equiv \sigma _0h_0^3/(3\mu )$. Importantly, the negative (positive) sign of the source term in Eq. (2) indicates that local regions of higher (lower) temperature lead to a local decrease (increase) in TLD film thickness.

### Coupling of SPP into grating covered with dielectric

Grating coupling is a common method to evanescently excite SPPs from free-space modes (see ref. ^10^ and references therein). Assuming that a plane wave with a wave vector component parallel to the grating, $\vec \beta _I$, is made incident on a metal surface with a grating of period Λ gives rise to a series of diffracted waves with wave vectors given by $\vec \beta _I + N\vec \beta _G$, where $\vec \beta _G = \left( {2\pi /{\mathrm{\Lambda }}} \right)\hat k_x$ is the grating vector that is perpendicular to the grating grooves (aligned along the *y*-direction, as described in Fig. 1) and *N* is the diffraction order. The diffracted waves along the interface can couple to an SPP mode provided the following momentum balance equation holds:^6^

3 $$\beta _I + N\beta _G = {\mathrm{Re}}\left( {\beta _{SPP}} \right)$$

where *β*_*I*_ = *k*_0_sin(*θ*), and $\beta _{SPP} = k_0\sqrt {{\it{\epsilon }}_m{\it{\epsilon }}_d/({\it{\epsilon }}_m + {\it{\epsilon }}_d)}$ is the SPP momentum^6^. Here, *θ* is the resonance coupling angle of incoming light, *k*_0_ = 2*π*/*λ* is the magnitude of a free-space wavenumber vector of wavelength *λ*, and *ϵ*_*m*_ and *ϵ*_*d*_ denote the dielectric constants of the metal and dielectric, respectively. Inserting the definitions of *β*_*I*_, *β*_*G*_, and *β*_*SPP*_ into Eq. (3) yields the following expression for sin(*θ*):

4 $${\mathrm{sin}}(\theta ) = \sqrt {\frac{{{\it{\epsilon }}_m}}{{{\it{\epsilon }}_m + n_d^2}}} - \frac{{\lambda N}}{{{\mathrm{\Lambda }}n_d}}$$

where $n_d = \sqrt {{\it{\epsilon }}_d}$. Treating the TLD film and air bilayer as a single medium with a depth-averaged index, obtained by integrating the index distribution along the direction normal to the metal surface^24^, we learn that the resonant coupling angle is a strictly decreasing function of the TLD film thickness. In particular, as the TLD film thickness increases in the range 0 ≤ *w* < *w*_*c*_, the resonant coupling angle tends to a normal incidence condition at critical thickness *w*_*c*_. For higher thickness values, i.e., for *w* > *w*_*c*_, the coupling angle becomes negative, which corresponds to a transition from the counter-propagating to the co-propagating regime of the coupled SPP mode (see Fig. S1 in Supplementary Material). To obtain a quantitative model that predicts the TLD film thickness (without approximations such as the depth averaging mentioned above) based on an experimentally measured resonant coupling angle, we turn to a numerical simulation (using Lumerical FDTD). While in real-space imaging, the resonant coupling angle coincides with the predetermined illumination angle, in *k*-space imaging, the resonant coupling angle can be inferred from the distance between the dark arcs, *D*, shown in the representative experimental images of Fig. 1e, f, via

5 $$\theta = {\mathrm{sin}}^{ - 1}(D/2k_0)$$

where *D* and *θ* can be functions of time due to thickness changes of the TLD film.

## Results

### Experimental setup

We employ two different laser sources, the so-called heating and probing beams described in Fig. 1, which are coupled to the same optical path, and deliver them to the sample. An argon laser (*λ* = 488, 514 nm) is used as the heating beam, which heats the metal substrate by optical absorption of the projected pattern and invokes TC flows accompanied by deformation of the TLD film; an NIR laser diode (*λ* = 785 nm) is used as the probing beam, which, upon reflection, provides an angular and spatial map for resonant SPP coupling. For a case of large deformations of the TLD film, which are within the reach of standard white-light microscopy, we employed a similar experimental setup, with a *λ* = 532 nm heating beam. An epi collection microscope was used to deliver both beams and image the fluid response (Olympus). The heating beam was collimated, optionally passed through a beam-shaping or transparency element, and then passed through a ×50 microscope objective (Mitutoyo) and focused to a point with a 1.5 μm width (FWHM). The probing beam was used to excite SPPs along the grating and was placed onto the heating beam path by using a dichroic mirror. The probing beam was delivered to the microscope objective in one of two configurations: collinear with the pump beam as a plane wave and focused on the sample (real-space imaging) and focused on the objective BFP and delivered to the sample as a plane wave (k-space imaging), both described in Fig. 1d and Fig. 1g, respectively. In the first configuration, the probing beam was then epi collected, and the BFP was imaged to measure the angular spectrum up to the objective numerical aperture, where the input angles satisfying the SPP momentum conditions appear as absorption lines in the angular spectrum. Changes in the momentum condition owing to the thickness change due to TC and healing flows were visualized as shifts in the resonance angle. In the second configuration, the collimated probing beam was passed through a lens (f = 40 cm) on a lateral translation stage and focused onto the BFP for Köhler illumination. Lateral displacements of the lens shifted the focus across the BFP, thus shifting the plane wave angle of incidence at the grating. The collected light was imaged by the microscope tube lens for direct observation of the grating surface, and regions with TLD film thickness matching the SPP condition at a given angle of incidence could be observed to darken according to resonant absorption. Because of the large thickness range in the fluid layer after the TC flow, sweeping of the illumination angles allowed visualization of the resonant contours of the corresponding level regions with thicknesses supporting the SPR resonance at the given angle. To form the TLD film, silicone oil of refractive index 1.39 was spun onto the fabricated gold grating (see Supplementary Material for more details).

### Model for TLD film thickness determination

**Fig. 2:**
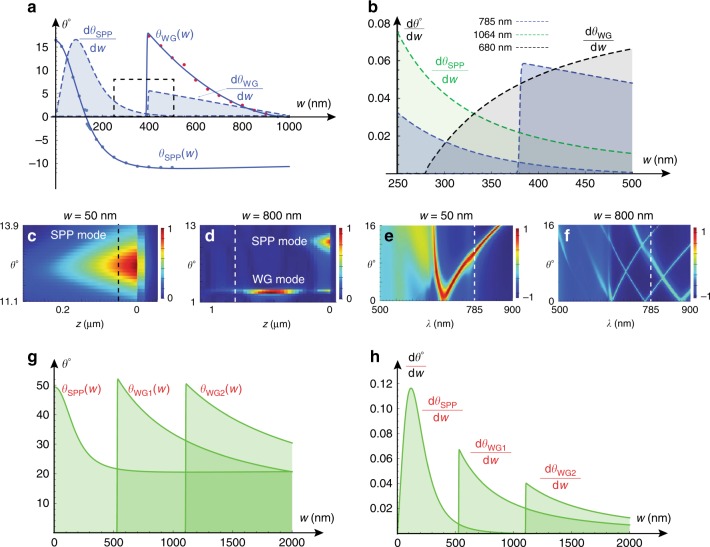
Numerical simulation results that relate the resonant coupling angle *θ* to the TLD mean film thickness *w*. **a** Discrete values and smooth interpolating curves, *θ*_*SPP*_(*w*) and *θ*_*WG*_(*w*), for *λ* = 785 nm, accompanied by the corresponding normalized sensitivity curves (filled). **b** The sensitivity curves of SPP and WG modes for *λ* = 1064 nm and *λ* = 680 nm, respectively, around the region of lower sensitivity for *λ* = 785 nm (dashed black box in (**a**)). **c**, **d** Electrical field intensity colormap in a *θ*-*z* plane, with dashed lines indicating the corresponding thickness values of *w* = 50 nm and thicker (*w* = 800) nm films. **e**, **f** The corresponding dispersion relation. **g** The *θ*_*SPP*_(*w*), *θ*_*WGI*_(*w*), and *θ*_*WG*2_(*w*) curves for a *λ* = 1064 nm probing beam and **h** the corresponding sensitivity curves

Extracting the TLD film thickness from experimental measurements requires a theoretical model that relates the thickness of the TLD film to the resonant coupling angle of the substrate guided modes. Figure 2 presents numerical simulation results of an incident plane wave coupling into propagating SPP and WG modes on a metal grating covered with a TLD film by using Lumerical FDTD. The key components of the simulation domain, presented schematically in Fig. 1a, consist of an incoming plane wave of wavelength *λ* = 785 nm, grating period Λ = 600 nm, and grating depth *q* = 30 nm. The dielectric constant of gold at 785 nm and the refractive index of silicone are taken as *ϵ*_*m*_ = −22.85+1.4245*i*^25^ and *n*_*d*_ = 1.39, respectively (see Supplementary Material for additional details). Figure 2a presents the resonant coupling angle curves *θ*_*SPP*_(*w*) and *θ*_*WG*_(*w*) into an SPP mode and into a higher-order WG mode (which emerges at higher thickness values^26^), respectively, as a function of the TLD film thickness *w*.

Importantly, both are strictly decreasing functions of *w* and therefore can be used to construct an inverse function, where *w* can be deduced based on the values of *θ*. The regions of positive and negative values of the function *θ*_*SPP*_(*w*) correspond, respectively, to SPP modes that propagate in a counter- and co-propagating direction relative to the in-plane component of the incident plane wave. These two different regimes are separated by an angular band gap centered at the normal incidence angle (see Supplementary Material section), which is reminiscent of the band gap observed near-normal incidence conditions on metal gratings without a dielectric layer^27^ due to destructive interference of the left- and right-propagating SPP modes. The filled curves in Fig. 2a present the corresponding sensitivity for the SPP and the higher-order WG mode, defined as *dθ*_*SPP*_(*w*)/*dw* and *dθ*_*WG*_(*w*)/*dw*, respectively. While the SPP mode admits the highest sensitivity value for a film thickness of ~100 nm and practically vanishes for thickness values above 600 nm, the first WG mode (which does not emerge at low thicknesses) admits the highest sensitivity at ~400 nm. These differences in regions of maximal sensitivity stem from the fact that, while for sufficiently thin films, the incoming plane wave couples to an SPP mode that admits the highest optical intensity on the metal surface (see Fig. 2c), for thicker films, the incident plane wave couples into a higher-order WG mode with the highest optical intensity at some finite distance from the metal surface within the TLD film, as presented in Fig. 2d (see also ref. ^28^ for optical intensity distribution along the vertical direction in dielectric-loaded SPP waveguides). Interestingly, the sensitivity of the WG modes drops in close proximity to the cutoff thickness values (i.e., film thickness that satisfies the WG cutoff condition^29^), seen around *w* = 375 nm in the center of the dashed region in Fig. 2a. As presented in Fig. 2b, one could potentially mitigate these lower-sensitivity regions by employing either a longer wavelength probing beam, which can couple to an SPP mode, or alternatively a shorter wavelength probing beam, which can couple to a WG mode, without modifying the grating properties.

Figure 2e presents the dispersion relation of the basic SPP mode, which is coupled to the metal grating that hosts a *w* = 50 nm dielectric. In particular, it shows a single curve, where the right and left branches correspond, respectively, to counter- and co-propagating modes relative to the in-plane component of the incident plane wave. The dashed vertical line at *λ* = 785 nm, which corresponds to the wavelength of the probing beam, intersects with the right branch, indicating that the given conditions facilitate coupling only to the counter-propagating SPP mode at a resonant coupling angle of 12.28°. Since the dispersion relation of WG modes propagating in a slab WG (formed, in our case, by the TLD film) is set by the constructive interference condition^29^, it follows that additional higher-order WG modes must emerge at even higher thickness values of the TLD film. Indeed, Fig. 2f presents an additional higher mode that emerges in thicker films of thickness 800 nm. The dashed vertical line of the probing beam at *λ* = 785 nm intersects with a left (co-propagating) branch of the SPP curve at 11.12° and with the right branch of the first WG mode at 2.51°.

Figure 2g presents the resonant coupling angle as a function of the film thickness for the SPP mode as well as for the first and the second WG modes for the case of a longer wavelength (*λ* = 1064 nm) probing beam, whereas Fig. 2h presents the corresponding sensitivity curves. Similar to the case of the *λ* = 785 nm probing beam described above, thicknesses around the cutoff condition of each one of the WG modes are accompanied by lower sensitivity regions. As expected, employing a higher wavelength allows extending the sensitivity range for higher film thicknesses due to the higher penetration depth of the corresponding SPP mode into the dielectric region; for instance, a sensitivity of 0.03°/nm can be achieved by employing a 785 nm wavelength at a thickness of 713 nm or by employing a 1064 nm wavelength at thicknesses of 872 nm or 1291 nm. It is instructive to mention that, in our work, the SPP and WG modes admit more than an order of magnitude higher sensitivity than that of the FP (Fabry-Perot) reflectance commonly used in white-light microscopy (see Supplementary Material).

### Dynamical optical manipulation of TLD films

**Fig. 3:**
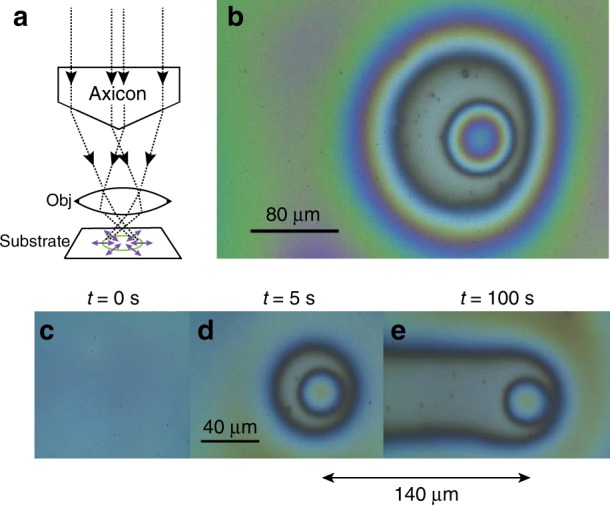
White-light microscopy experimental images of optically driven droplet formation and translation along the substrate. **a** Ray-path scheme presenting refraction from the axicon’s conical surface, the ring-shaped illumination pattern on the substrate and the direction of mean TC flow (blue arrows). **b** Optically induced droplet of 60 μm in diameter from a TLD film of thickness 400 nm due to a 400 mW laser power source of wavelength 532 nm operating for 30 s (×20 objective). The asymmetry of the annular-shaped deweted region stems from slight asymmetry in the illumination pattern. **c**, **d**, **e** Time sequence of the formation of a droplet with a diameter of ~30 μm by applying a similar laser source to a 200 nm thick TLD film and a ×50 objective; **c** prior to illumination *t* = 0 s, **d** *t* = 5 s, and **e** *t* = 100 s, and a 140 μm horizontal substrate translation

To probe optical control over the shape of the liquid-gas interface, we employed a novel optical patterning system to deliver light illumination through a beam-shaping element. The delivered light pattern induces a thermal gradient on the liquid-gas interface and consequently triggers TC flows, which allow manipulation of its shape. In particular, fixing an axicon beam-shaping element to the Fourier plane of the objective lens transforms the substrate plane focal point into a focused ring-shaped illumination pattern on the substrate (Fig. 3a) and leads to TC flows that invoke film thinning in the regions of highest temperature. Figure 3b presents a white-light microscopy image of the generated droplet of ~60 μm in diameter from a 400 nm thick silicone oil film, spun on a NiCr substrate, due to a 400 mW laser source of wavelength 532 nm and an illumination time of 30 s. Film deformations were measured using a white-light reflection microscope (Leica, ×50) and a ×20 objective. Figure 3c-e present droplet formation from a thinner 200 nm thick silicone oil film and droplet translation along the metal substrate, achieved by translating the microscope stage and keeping all other components of the optical setup stationary. In this case, we utilized a higher magnification objective, ×50, which resulted in a higher optical power and consequently shorter illumination time of 5 s. The speed of stage translation in our experiments was ~1.4 μm/s, which was sufficiently low to ensure that the invoked TC flows in the silicone oil film around the droplet led to a ring-shaped rupture as the optical pattern was progressively translated along the substrate. For a fixed optical power and faster substrate translation rates, the droplet size was progressively reduced. Interestingly, since the healing time of the silicone oil film due to the formed rupture was longer than the translation time of the substrate, a wake behind the droplet was formed.

While for the analysis of optically thick TLD deformations, white-light contrast is sufficient, high-sensitivity characterization is much more challenging for processes where the resultant TLD film thickness variations are small. Below, we apply the SPRM method coupled to our optical manipulation system to demonstrate much higher sensitivity.

### *k*-space imaging

**Fig. 4:**
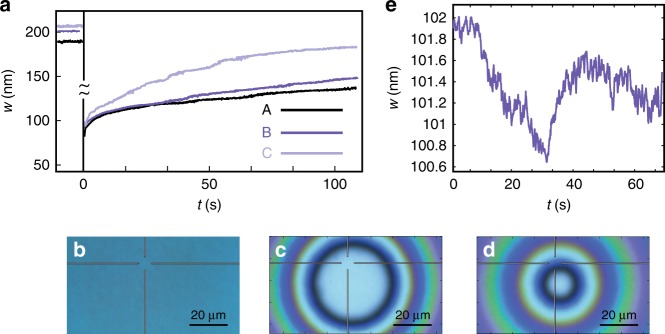
Subnanometer measurements of the dewetting and healing dynamics of thin liquid film via the *k*-space method, and white-light microscopy imagesof the formed rupture. **a** *k*-space imaging: TLD film thickness as a function of time at the center of a focused heating beam (beam waist of diameter 0.62 μm) for three different optical powers: (A) 34.57 mW, (B) 17.96 mW and (C) 1.79 mW; the corresponding intensities on the sample are given by (A) $1.14 \cdot 10^{11}$ W/m^2^, (B) $5.94 \cdot 10^{10}$ W/m^2^ and (C) $5.92 \cdot 10^9$ W/m^2^. We attribute the difference in film thickness between different experiments at times *t* < 0 to spontaneously formed nonuniformity of the free interface. **b**, **c**, **d** Top view of white-light microscopy images of TLD film rupture under optical power employed in case A, captured at three moments in time: **b** before optical heating, **c** 10 s after the heating is switched off and **d** 30 s after the heating is switched off. (**e**) Film thickness as a function of time on a subnanoscale due to 20 s of illumination of a much less intense beam of intensity $1.98 \cdot 10^7$ W/m^2^ (power 6 μW) and TLD film healing after the beam is switched off at *t* = 30 s. In particular, the silicone oil thickness change during the period from 30 s to 35 s is from ~100.6 nm to ~101.2 nm, which is ~0.6 nm

Figure 4a presents the experimental results of silicone oil film thickness as a function of time at the illumination spot due to three different optical heating powers. Prior to applying the laser heating beam (*t* < 0), all three experiments show nearly constant thickness values, whereas at later times (0−20 s), the fluid thickness is reduced due to the TC flows, which carry the fluid towards regions of lower temperature away from the focused illumination spot. As expected, higher optical powers lead to thinner films; however, in cases *A* and *B* of highest optical intensity, the corresponding thickness as a function of time shows a very similar behavior. This similarity is indicative of a rupture process in the silicone oil film that exposes disk-shaped regions on the substrate, as shown in the white-light microscopy images in Fig. 4b–d, for a similar optical power and thicker (0.5 μm) silicone oil film. Specifically, since the rupture expansion rate due to a heating source of fixed intensity progressively decreases as the rupture grows, similar to the thinning rate of optically stimulated, significantly thicker fluid films^30^, we expect to obtain similar ruptures in cases in which the total illumination time is significantly larger than the rupture formation time. After the heating beam is switched off, all curves *A*–*C* show an increase in film thickness, which is indicative of the healing process due to the capillary forces that eventually lead to a nearly flat film.

Figure 4e presents the thickness measurement of silicone oil at much lower optical power (6 μW), which allows the capture of its dynamics during the operation of the heating beam. Specifically, during the operation of the heating beam from 10−30 s, the silicone oil film shows a decrease in its thickness, whereas at later times after the heating beam is switched off (*t* > 30 s), it shows a healing process characterized by an increase in its thickness, qualitatively similar to the healing process occurring at higher powers described in Fig. 4a. Notably, the thickness change of the silicone oil film occurs at a subnanometer scale but still over a long period of time, which is on the order of seconds. This large characteristic healing time scale allows ruling out other thermally driven effects, such as the thermo-optical effect, which is governed by heat diffusion processes and therefore operates on a much shorter time scale on the order of several microseconds (see Supplementary Material). Moreover, even for the case where the thermo-optical effect has a nonnegligible effect, it would still be much smaller than the corresponding contribution due to TLD film deformation driven by the TC effect under identical optical power^14^.

### Real-space imaging

**Fig. 5:**
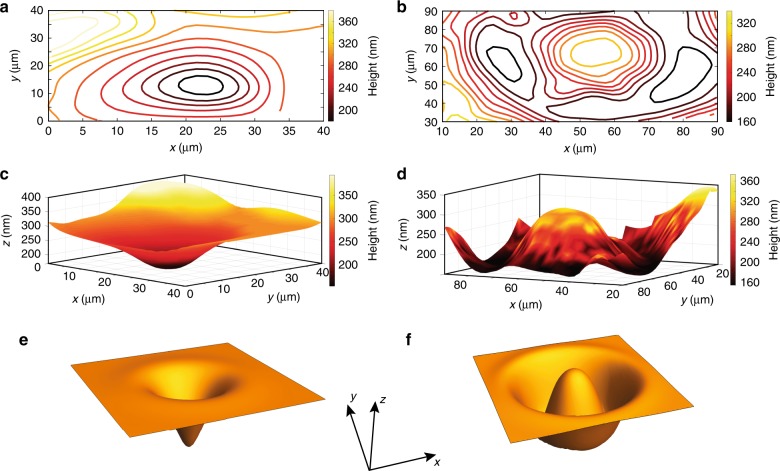
Real-space topography measurements of optically induced indentation and droplet in thin liquid film. Real-space imaging experimental results presenting contour maps (**a**, **b**) and interpolated 3D topography maps (**c**, **d**) of silicone oil film due to focused (**a**, **c**) and ring-shaped (**b**, **d**) illumination patterns. **e**, **f** Numerical simulation results of TLD film deformation (Eq. (2)) for focused (**e**) and ring-shaped (**f**) illumination patterns. The size of the domain along the in-plane *y*-*z* directions is 20 × 20, and the maximal value of the deformation is 0.06 in dimensionless units (see more details in Supplementary Material)

Figure 5 presents the experimental results of the TLD film equal-height contour lines as well as the corresponding interpolated topography maps. In particular, it shows an indentation formed under focused illumination in a small region with a diameter of approximately 0.65 μm (Fig. 5a, c) and a droplet formed under a ring-shaped region with an inner diameter of ~40 μm (Fig. 5b, d). These optically induced heat patterns, which lead to different fluidic patterns, are qualitatively captured by numerical simulation of the corresponding cases presented in Fig. 5e, f. The dark resonant contours presented in Fig. 5a, b correspond to different equal-height contours of the TLD film free surface, obtained by sweeping the corresponding incident angles and capturing the reflected light from the sample. The step in the illumination angle sweep is 0.77° per step, and then, for each value of the resonant coupling angle, we used the theoretical curve to determine the thickness of the silicone oil film.

After the heating beam is switched off, the thin film healing rate is relatively fast, as could also be observed via *k*-space imaging in Fig. 4a just after *t* = 0 s. The latter implies that real-space imaging of dynamical processes is applicable to processes on time scales smaller than the time scale required to scan the relevant angles. In the present setup, the scanning time was 10 s; this consideration dictated performing the angle scan at later times, characterized by a slower healing process, where the droplet already coalesced with the surrounding thin film.

It is instructive to mention that the curvature of the TLD film in our experiments was sufficiently low, and therefore, its effect on the coupling angle is negligible. Indeed, as seen from Fig. 5c, the mean slope is approximately Δ*z*/Δ*x* = 100 nm/20 μm = 5 × 10^−3^, which is smaller than the critical value 8 × 10^−2^ predicted by the analysis from Fig. S2(c, d) (see Supplemental Material for details).

### Effect of thin film periodic undulation on the coupling angle

**Fig. 6:**
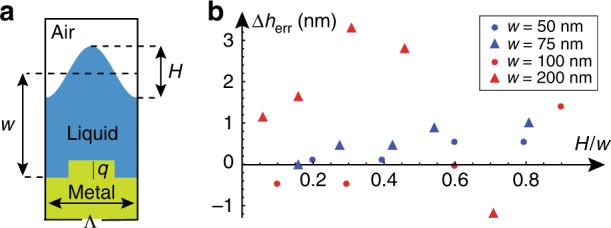
Numerical results presenting the effect of periodic undulation of thin dielectric film surface on the predicted values of its thickness. **a** Numerical simulation domain presenting a basic unit cell of period Λ = 600 nm and grating depth *q* = 30 nm that hosts a symmetrically undulated TLD film of the same period, with peak-to-peak amplitude *H* and mean thickness *w*. **b** Simulation results of the predicted error of the film thickness, Δ*h*_err_, relative to a flat TLD film with the same mean thickness and *H* = 0. In particular, **b** presents Δ*h*_err_ as a function of the normalized deformation *H*/*w* for mean thicknesses *w* = 50,75,100,200 nm

We now analyze the effect of possible periodic undulation of the TLD film surface due to an uneven subwavelength coupling grating (see Fig. 6a) on the resonant coupling angle. The governing equation for the thickness of a thin Newtonian liquid film (note that the silicone oil employed in our experiments is a Newtonian fluid) under the effects of surface tension and centrifugal forces due to rotation with angular velocity *ω* is given by^31^

6 $$\frac{{\partial ^3\eta }}{{\partial x^3}} + {\mathrm{\Omega }}^2[1 - \left( {\frac{w}{{w - q}}} \right)^{ - 3} + 3\left( {\frac{w}{{w - q}}} \right)^{ - 4}\eta ] = 0$$

where ${\mathrm{\Omega }}^2 \equiv \rho \omega ^2({\mathrm{\Lambda }}/2)^3r_0/(\sigma (w - q))$, and a similar equation with *q* = 0 holds over grooves. Here, *w*−*q* represents film thicknesses above a grating of depth *q*, *r*_0_ is the distance from the rotation axis, and Ω^2^ represents a dimensionless ratio of centrifugal to capillary forces. Inserting the following parameters relevant to our system: *ρ* = 970 kg m^−3^, *ω* = 100 s^−1^, Λ = 6 × 10^−7 ^m, *r*_0_ = 10^−3^ m, *σ* = 10^−3^ N m^−1^, and *w*−*q* = 5 × 10^−8^ m, into the definition of Ω^2^ given by Eq. (6), we learn that ${\mathrm{\Omega }}^2 = 2.06 \cdot 10^{ - 4}$ ≪ 1. The latter implies that the conditions in our system correspond to a surface-tension-dominated regime, which leads to a practically planar fluid-gas interface (see^32^ for a case where Ω^2^ ≈ 10^−2^). Indeed, we find that TLD films of thickness values in the range between *w* = 50 nm and 270 nm acquire a peak-to-peak undulation depth *H* on the order of 10^−5^–10^−7^ in units of *w*−*q*, i.e., much smaller than 1 nm (see Supplementary Material for more details). Nevertheless, we note that higher values of *H* can, in principle, emerge under different experimental conditions that are not met in our work, such as higher values of Ω^2^ or cases where non-Newtonian fluids or complex fluids with volatile components (e.g., polymers or photoresists) are employed^33^. Figure 6b presents numerical simulation results of the error in the TLD film thicknesses *w* = 50,75,100,200 nm due to the presented values of the undulation peak-to-peak amplitude *H* (in units of *w*). Interestingly, even large values of *H*/*w* lead to a very small shift in the resonant coupling angle. For instance, for the case where *w* = 50 nm and *H* = 30 nm (which is 60% of the mean thickness), an error is introduced around Δ*h*_err_ = 0.5 nm, which corresponds to only 1.5% of the mean thickness *w* (see Supplemental Material for more details). Importantly, combining the small error introduced by the symmetric undulation amplitude *H* with the analysis above indicating that *H* is expected to be smaller than 1 nm, we learn that the corresponding value of Δ*h*_err_ is expected to be much smaller than 1 nm even in the presence of a 30 nm deep grating.

## Discussion

In this work, we presented dynamical optical patterning of a TLD film and two novel plasmonic methods for the measurement of its thickness and topography, which were applied to determine the film dynamics after being driven out of equilibrium by an optically induced TC effect. In particular, we leveraged the TC effect as a novel heat-based mechanism to induce tuning of the plasmonic resonances. The imaging methods presented in our work are complementary in the sense that while *k*-space imaging provides thickness values as a function of time in a small region, real-space imaging is applicable to a larger area of a few hundreds of microns. Both methods admit immediate generalizations that can be utilized to probe specific fluidic processes by introducing higher-speed sweeping mechanisms (e.g., translation stages) and higher-frame-rate cameras; *k*-space imaging can be extended to provide thickness information along spatial segments that can cover 2D regions, whereas real-space imaging can, in principle, capture faster processes that evolve over shorter characteristic times. Our methods open a door for future studies where thickness changes of the TLD film monitored at a subnanometer scale would be indicative of various physical processes, such as thermocapillary instabilities in polymer molten films^34^ and instabilities due to electrohydrodynamic^35^ and electrostatic^36^ effects. In addition, the methods are applicable to studying pore nucleation^37^, phase transitions in thin polymer films^38^ and light-induced reversible thickness changes of photosensitive materials^39^ without the need to employ, respectively, AFM imaging, X-ray reflectivity, and neutron reflectometry methods. Importantly, our methods are not compromised if an additional thin layer of solid dielectric is deposited on top of the metal grating to achieve desired surface properties, which, in principle, allows studying basic problems, such as the wetting and spreading of thin films on different substrates. Our analysis of the effect of fluid-gas interface periodic undulation on the resonant coupling angle, due to possible interaction with a nonuniform topography (metal grating), showed that it introduces a negligible error in the predicted thickness values. Nevertheless, periodic undulation could be of importance in future studies involving non-Newtonian fluids with volatile components (e.g., polymers), where the peak-to-peak undulation depth may be larger and asymmetrical. Furthermore, we demonstrated a novel optical method/process to create and translate droplets directly from a TLD film, which merits further investigation from a fluid dynamical perspective and may also be useful for future applications in microscopy/nanoscopy^40^, where each droplet can serve as a smooth, tunable and mobile micro/nanolens, and in bio-oriented applications in microfluidics. In fact, since the temperature increase required to form the droplets from the liquid is expected to be only a few degrees or less (see Supplemental Material), we expect that our method may be suitable for various applications in the biosciences, such as single virus communication and PCR (polymerase chain reaction) assays, where compartmentalization of the TLD film into an array of droplets may be useful to invoke independent biomolecular reactions^41^. We hope that our work, which brings together plasmonic effects, SPRM and fluid dynamics, will stimulate the future development of specific optical systems aimed at studying TLD film processes and light-fluid interactions. In particular, other prominent SPRM variants that can be employed include the classical Kretschmann configuration to study the dynamics of TLD films of thicknesses below the grating depth, the holographic-based method^42^, which offers the possibility of simultaneous detection of reflectivity and phase changes in SPR images, and SPP confocal interferometry^43^, which offers superior noise performance and can improve the lateral resolution.

## Materials and methods

For the TLD film substrate, which supports SPP excitations, we employed gold gratings with a period of 600 nm, a 50% duty cycle, and grooves of 30 nm in depth, which were fabricated by nanoimprint pattern transfer and lithography. Using electron-beam evaporation, a silicon substrate was layered with 5 nm of Ti as adhesion and 200 nm of Au to form the grating bulk material. A nanoimprint resist bilayer was spun and soft-baked on top, with 75 nm of PMMA forming the underlayer and 160 nm of upper layer resist (AR-UVP), onto which a polymeric mold containing the grating features was aligned and stamped while curing the imprinted resist (EVG aligner). After mold/sample separation, reactive ion etch (RIE) recipes were used to remove residual top and bottom layer resists within the pattern trenches. The pattern was transferred from imprint resist to a metal mask by 30 nm of Cr deposition and acetone liftoff. Using the Cr mask, an additional RIE recipe was used to directly remove 30 nm of Au within the exposed trenches, followed by wet etch removal of the Cr. The pattern depth, duty cycle, and periodicity were confirmed by AFM measurements (Nanonics).

For droplet generation in the white-light imaging setup, we used a 25.4 mm diameter Axicon (Thorlabs AR coated UC fused silica) with a deflection angle of 0.23° and a reflectance less than 1% in the range of 350–700 nm.

To form a TLD film, silicone oil (Fisher Scientific) of refractive index 1.39 was spun onto the grating by repeated intervals of spin coating at 10,000 rpm. The baseline thickness of the prepared fluid film was measured by spectral reflectance to be on average 176 nm by a separate optical profilometer (Filmetrics F20). A complete spin curve for 3–12, with each spin being 1.5 min in duration, was obtained to prepare baseline average fluid thicknesses from 175 to 700 nm.

The two complementary imaging modalities we employed in this work, i.e., real-space imaging and *k*-space imaging, employ a very similar optical design, described by Fig. S4 in Supplementary Material. In both imaging methods, the low-power *λ* = 785 nm probing beam and the higher-power *λ* = 488, 514 nm heating beam were brought to the same optical path by means of a shortpass filter (SPF). Upon reflection from the sample, the light was imaged to a CMOS camera by utilizing a 50:50 beam splitter (BS). Common to both imaging techniques is a microscope imaging system with a long working distance, a ×50 objective and an internal tube lens. Real-space imaging was accomplished by placing the CMOS camera at the focal plane of the tube lens. For *k*-space imaging, we employed additional lenses to image the BFP (see Supplementary Material for more details).

The standard figure of merit for sensitivity definition is to take it as three times sigma, where sigma is defined as the standard deviation of the signal. In our case, it is natural to compute sigma as the standard deviation of the fluctuating signal as a function of time, with the heating beam switched-off and the probing beam switched-on; direct computation yields sigma equal to 0.2 nm, and the resultant sensitivity is then 0.6 nm.

## Supplementary information

Supplemental Material

k-SPACE MEASUREMENT OF TLD FILM THICKNESS DUE TO TC FLOWS AND HEALING PROCESS
