# Supplementary material for: Subnanometer imaging and controlled dynamical patterning of thermocapillary driven deformation of thin liquid films
Source: Light Sci Appl. 2019 Aug 28;8:77. doi: 10.1038/s41377-019-0190-6 (PMC6804570; doi:10.1038/s41377-019-0190-6)
Supplement: Supplementary file 1 — Supplemental Material [file 41377_2019_190_MOESM1_ESM.docx]

##### Supplementary Material for “Subnanometer imaging and controlled dynamical patterning of thermocapillary driven deformation of thin liquid films”

Shimon Rubin, Brandon Hong, Yeshaiahu Fainman

Department of Electrical and Computer Engineering, University of California, San Diego, 9500 Gilman Dr., La Jolla, California 92023, USA

## 1 Momentum conservation diagrams

Fig. S1 presents schematic description of the momentum conservation relation, given by Eq.3 in the main text. The latter describes coupling of an incident light into an SPP mode of momentum $\beta_{SPP}$, and allows to deduce the corresponding resonant coupling angle $\theta.$ In particular, employing Eq.5 in the main text as well as the mutual orientation of the emerging two dark resonant arcs, allows to determine $\theta$ by experimental measurement of the distance between the arcs, $D$. For films thinner than the critical thickness $w_{c}$, i.e. for $w<w_{c}$, the emerging resonant dark arcs point towards each other (Fig. S1b), whereas for thicker films, i.e. for $w>w_{c}$, the dark arcs point in the opposite directions (Fig. S1d). At critical thickness, i.e. at thickness which leads to $\theta=0$, there is a transition between the two dark arcs orientation regimes. Furthermore, while in the $w<w_{c}$ regime the in-plane component of an incident free-space mode is anti-parallel to $\vec{\beta}_{SPP}$ ($\theta>0$), the $w>w_{c}$ regime is characterized by $\theta<0$ and SPP mode momentum which is parallel to the in-plane component of an incident plane-wave momentum. Importantly, some cases such as the $\lambda=1064$ nm probing-beam incident on a 600 nm periodicity gold grating, described in Fig. 2g in the main text, do not admit critical height $w_{c}$. Consequently, the relative orientation of the emerging dark arcs in such cases is oriented towards each other for all thickness values. For sufficiently thick film, above a WG threshold condition, the dielectric film and the metal grating support also resonant coupling into WG modes. The corresponding resonant angle diagrams would then admit an additional pair of dark arcs (one pair for each higher order WG mode), on top of the dark arcs associated with the SPP mode. Small thickness perturbations of such thick films would lead to changes of the WG dark arcs whereas the SPP resonant curves would not change appreciably, as could be also anticipated from the low sensitivity regions of SPP curves at high film thicknesses, presented in Fig. 2 in the main text.


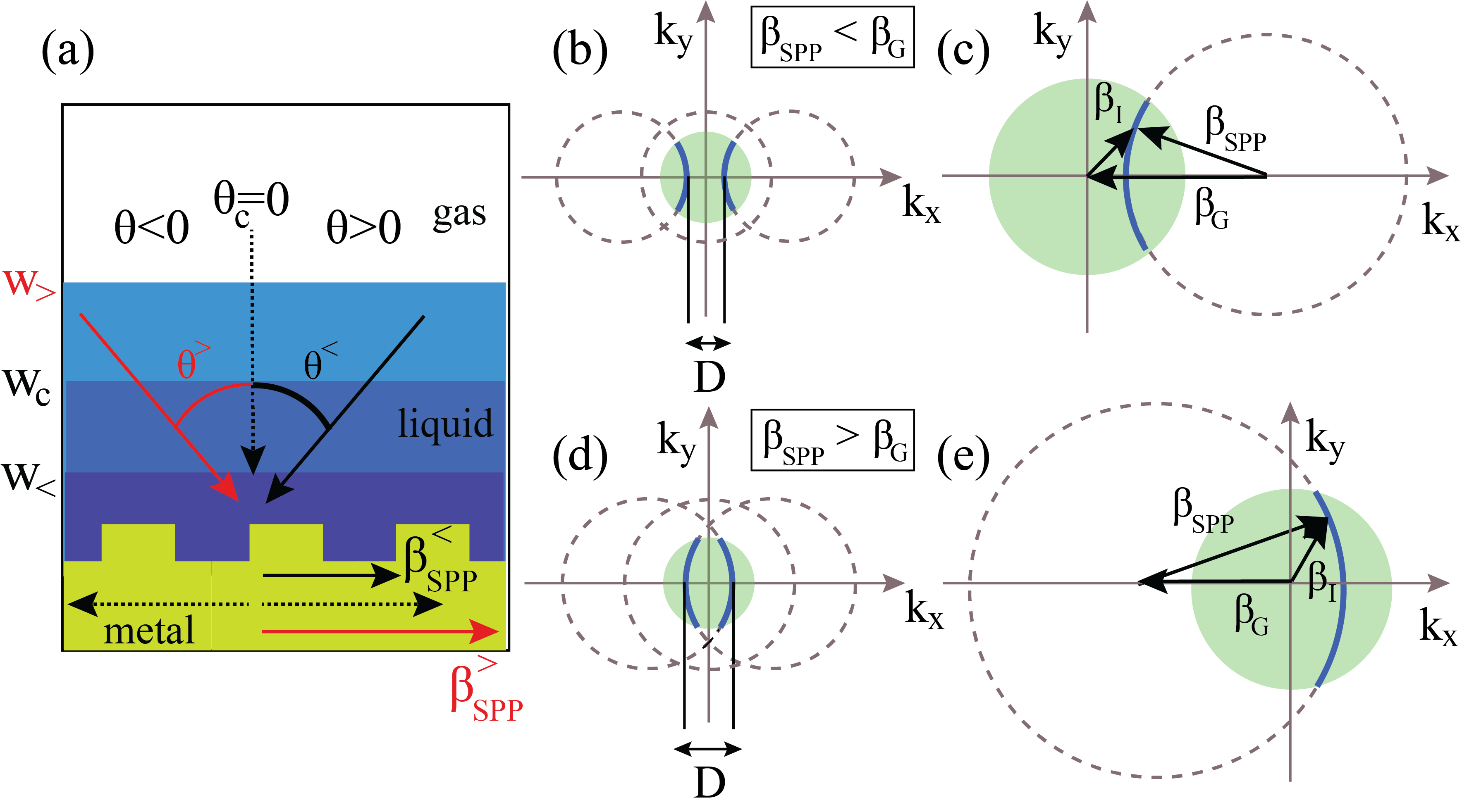


Fig. 1: (a) Coupling regimes of an incident plane wave into SPP mode which propagates on a metal grating covered with a dielectric film; thin film regime for thicknesses $w<w_{c}$, and thick film regime for thicknesses $w>w_{c}$, where $w_{c}$ is the critical thickness which corresponds to the normal angle coupling. (b,d) Present the allowed SPP dark arcs formed by an intersection of the set of incident light directions (green disks) with the shifted SPP momenta for the two regimes $\beta_{SPP}<\beta_{G}$ and $\beta_{SPP}>\beta_{G}$. (c,e) Present the momentum balance for the two regimes where the excited SPP mode carries in-plane momenta in the direction opposite (c) and parallel (e) relative to the in-plane component of the incident light momentum.

## 2 Underlying main assumptions of Eq.5

The free surface of a Newtonian liquid film of viscosity $\mu$ and stress tensor $\tau_{ij}$ satisfies the following stress balance equation [1]

$\tau_{ij}n_{j}=\sigma n_{i}\vec{\nabla}\cdot\hat{n}-\vec{\nabla}_{\parallel}\sigma; i,j=x,y,z,$ (1)

where $\sigma$ is the surface tension, $\vec{\nabla}_{\parallel}$ stands for a gradient with respect to the in-plane coordinates ($y,z$) and $\vec{\nabla}\cdot\hat{n}$ is the 3D divergence of the unit vector normal to the film surface. Assuming the surface tension depends linearly on the temperature via $\sigma(T)=\sigma_{0}-\sigma_{T}\Delta T$, applying lubrication approximation for the Navier-Stokes equation, taking the thin film limit of the matching conditions on the fluid-gas interface [1], and assuming quasi-static temperature field distribution, yields [3]

$\frac{\partial\eta}{\partial t}+D_{\sigma}\nabla_{\parallel}^{4}\eta=-\frac{\sigma_{T}w_{0}^{2}}{2\mu}\frac{\Delta T}{I_{0}d^{2}}\chi I.$ (2)

The minus (positive) sign of the source term in Eq.(2) indicates decrease (increase) of local thickness for positive (negative) Marangoni constant. Scaling to dimensionless variables via $t\to\tau_{l}t$ where $\tau_{l}=d^{4}/D_{\sigma}$, $\vec{r}_{\parallel}\to(\tau_{th}\cdot D_{\sigma}^{1/4})\vec{r}_{\parallel}$, $\eta\to w_{0}\eta$ and $I\to I/I_{0}$ yields

$\frac{\partial\eta}{\partial t}+\nabla_{\parallel}^{4}\eta=-\mathrm{Ma}\cdot\chi\cdot\frac{\tau_{l}}{\tau_{th}}I/2,$ (3)

where $\mathrm{Ma}\equiv\sigma_{T}\Delta Tw_{0}/(\mu D_{th}^{m})$ is the dimensionless Marangoni number.

The heat diffusion equation that governs the temperature field, $T^{m}$, on a metal substrate with heat diffusivity $D_{th}^{m}$ is

$\frac{\partial T^{m}}{\partial t}-D_{th}^{m}\nabla_{\parallel}^{2}T^{m}=\frac{\Delta T}{I_{0}\tau_{th}}\chi I; \chi\equiv\frac{\alpha_{th}^{m}d^{2}I_{0}}{k_{th}^{m}\Delta T}.$ (4)

The latter implies that the maximal temperature arise, $\Delta T^{max}$, due to a circular laser beam of intensity $I$ and waist $\mathcal{l}_{0}$, is subject to

$\Delta T^{max}=\frac{\alpha I\mathcal{l}_{0}}{k_{th}^{m}},$ (5)

where $\alpha$ is the optical absorption coefficeint. For a low power beam of intensity $I=19.98\cdot10^{7}$ Wm${}^{-2}$ and heat conductivity of gold, which is around $k_{th}^{m}=300$ W m${}^{-1}$K${}^{-1}$, reflectance at $\lambda=514$ nm and $\mathcal{l}_{0}=0.8$ $\mu$m, the corresponding $\Delta T^{max}=0.25$ K.

## 3 Derivation of Eq.6

The governing equation for the axisymmetric flow of a TLD Newtonian film under the effects of surface tension and centrifugal force due to rotation of the substrate with axisymmetric roughness, and furthermore under lubrication approximation is given by [4]

$r\frac{\partial h}{\partial t}=-\frac{1}{3\mu}\frac{\partial}{\partial r}\left( \sigma h^{3}r\frac{\partial^{3}(h+s)}{\partial r^{3}}+h^{3}\rho\omega^{2}r^{2} \right).$ (6)

Here, $r$ is the radial coordinate, $\rho$ is the fluid density, $\omega$ is the angular velocity, $h$ is the local fluid thickness relative to the topographic feature and $s$ is the local substrate height, so $h+s$ is the local height of the fluid-gas interface relative to the substrate. Switching to dimensionless variables via

$\begin{matrix} & R\equiv\frac{r}{w-q},X\equiv\frac{r-r_{0}}{w-q},H\equiv\frac{h}{w-q},S\equiv\frac{s}{w-q}, \\ & T\equiv\frac{t}{t_{c}},t_{c}\equiv\frac{\Lambda\mu}{2r_{0}\rho\omega^{2}(w-q)^{2}} \end{matrix}$ (7)

where $w-q$ represents fluid thickness above the grating ridge and $t_{c}$ is the time scale based on the centrifugal force, and furthermore dropping the time derivative, yields the following time independent equation for dimensionless thin film thickness [4]

$\left( \frac{\partial^{3}H}{\partial X^{3}}+\frac{\partial^{3}S}{\partial X^{3}} \right)H^{3}+\Omega^{2}H^{3}=\Omega^{2}; \Omega^{2}\equiv\frac{\rho\omega^{2}(\Lambda/2)^{3}r_{0}}{\sigma(w-q)},$ (8)

where $\Omega^{2}$ represents a dimensionless ratio of centrifugal to capillary forces. Here, dropping the time derivative is justified because the characteristic time for the film thinning process, $t_{s}\equiv2r_{0}t_{c}/\Lambda$, is much larger than that for the rotational flow $t_{c}$, and therefore we can expect the film profile over the grating to adjust to changes in the overall film thickness at a rate much faster than the overall film thickness itself changes [4]. In fact, in our system $\Lambda=6\cdot10^{-7}$ m, $r_{0}=10^{-3}$ m and $2r_{0}/\Lambda\gg1$. Treating to $S$ as a piece-wise constant function, and linearizing Eq.(8) above the grating ridge and trench via $H=1+\eta$ and $H=1+q/(w-q)+\eta$, respectively, we can omit the derivative of $S$ leading to [4]

$\frac{\partial^{3}\eta}{\partial x^{3}}+3\Omega^{2}\eta=0,$ (9)

$\frac{\partial^{3}\eta}{\partial x^{3}}+\Omega^{2}[1-\left( 1+\frac{q}{w-q} \right)^{-3}+3\left( 1+\frac{q}{w-q} \right)^{-4}\eta]=0.$ (10)

## 4 Details of numerical simulation

**Geometry**: The scheme of the simulation domain is presented at Fig. 1a in the main text and it resides in the region $-0.3$ $\mu$m $\leq x\leq0.3$ $\mu$m and $-3$ $\mu$m $\leq y\leq$ $1.5$ $\mu$m. The metal substrate resides in the region $-3$ $\mu$m $\leq y\leq0$ $\mu$m and it hosts another $30$ nm depth metal grating stripe in the region $-0.15$ $\mu$m $\leq x\leq0.15$ $\mu$m (i.e. $50$% duty cycle) and $-0.33$ $\mu$m $\leq y\leq-0.3$ $\mu$m.

**Computational domain**: The computational grid accommodates $67$ and $279$ elements along the $x$- and $y$-axis, respectively. While the spacing between numerical grid points is uniform along the $x$-axis, the grid is non-uniform along the $y$- axis. Tha latter admits a finer mesh near the metal surface, accommodating $35$ elements in the region near the metal surface, $-0.33\mu m\leq y\leq-0.3$ $\mu$m. The implemented boundary conditions are: Bloch along $x$-axis and PML along $y$ and $z$ axes. PML settings: stretched coordinate PML; min layer: 12; max layers: 64; kappa=3; sigma=1.5; polynomial:3; alpha=0; alpha polynomial:1.

**Dielectric properties:** For incident plane wave of wavelength $\lambda=785$ nm and $\lambda=1064$ nm, the corresponding dielectric constant of gold is $\epsilon_{m}=-22.855+1.4245i$ $\epsilon_{m}=-48.450+3.6006i$, respectively [5]. The refractive index of the thin film is $1.39$, which corresponds to refractive index of silicone oil employed in our experiments.

**Source and monitor:** The source is a plane wave injected at $y=-1.7$ $\mu$m at different angles, typically between $0$ and $20$ relative to the $y$ axis. The monitor collects the reflected wave at $y=-2.2$ $\mu$m.

## 5 Numerical results of an angular band-gap and the effect of thin film’s curvature on the resonant coupling angle

Increasing the dielectric thickness $w$ on top of the metal grating from zero thickness (i.e. no dielectric) to higher values, leads to a monotonic increase of the coupled SPP wavevector magnitude, and therefore results in an increasingly smaller resonant coupling angle. In some cases where the coupling angle at $w=0$ is sufficiently low, the dielectric angular band-gap opens around the normal incidence angle at critical thickness $w_{c}$, which results in a lower coupling efficiency into the corresponding SPP mode. Fig. S2 below, describes coupling of a TM polarized $785$ nm plane-wave into propagating SPP mode on a gold grating (grating period $600$ nm), and shows an angular bandgap around critical thickness, $w_{c}=133$ nm (Fig. S2b).


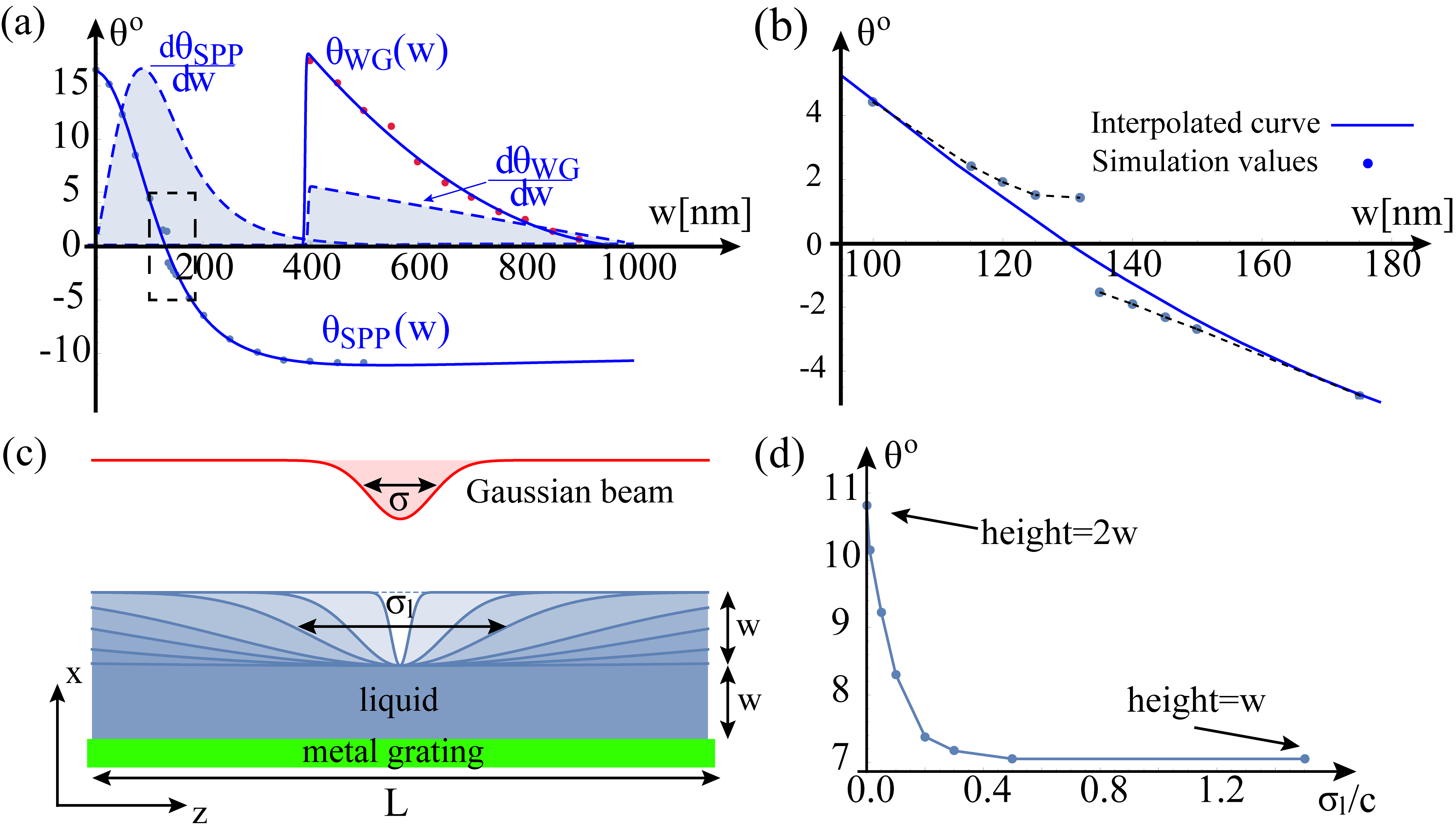


Figure 2: Numerical results presenting: (a) coupling angle curves to SPP and WG modes for probing beam of wavelength $\lambda=785$ nm and (b) angular band-gap formed around the normal incidence angle at critical thickness around 133 nm; (c) Surface topographies described by Eq.11 for the values $\sigma_{l}/c=0,0.01,0.05,0.1,0.2,0.3,0.5,1.5$, $L=21$ $\mu$m, $w=225$ $\mu$m, distance of source from the metal is $850$ nm, $\sigma=3$ $\mu$m, and $c=42\cdot10^{-6}$ dimensionless constant; (d) The resonant coupling angle of the Gaussian probing beam of foxed width $\sigma$, as a function of the indentation width $\sigma_{l}$. For sufficiently wide (narrow) indentations the coupling angle tends to the resonant angle of flat film of thickness $w$ ($2w$).

We would like to probe the effect of local non-homogeneity in TLD film thickness, on the value of the corresponding resonant coupling angle to the SPP mode. To this end, we construct a numerical simulation domain of width $L$, schematically presented in Fig. S2c, and employ a commercial-grade simulator based on the finite-difference time-domain method (Lumerical, FDTD). The shape of the non-homogeneity is taken as an indentation of a Gaussian form, with a width parametrized by $\sigma_{l}$ via

$x(z)=w[2-exp(-z^{2}/\sigma_{l}^{2})].$ (11)

Fig. S2d presents numerical results of the corresponding resonant angle as a function of indentation’s width, for a Gaussian-shaped incident light source, $exp(-z^{2}/\sigma^{2})$, of a fixed width at half maximum $\sigma$. In the wide indentation limit, described by $\sigma_{l}>\sigma$, the coupling angle tends to the resonant angle that corresponds to a flat dielectric of thickness $w$, whereas in the opposite narrow indendation limit ($\sigma_{l}<\sigma$), the coupling angle tends to the resonant angle that corresponds to a flat dielectric of thickness $2w$. In particular, we learn that for increasingly smaller values of $\sigma_{l}$ the coupling angle experiences a significant shift only at sufficiently sharp indentations that introduce a mean slope around the value $2w/L\simeq0.02$.

## 6 Numerical simulation details in Fig.4

Numerical integration of Eq.(3) is accomplished by utilizing commercial grade numerical solver (Mathematica, v. 11) and implementing its built-in Explicit Runge Kutta method. The size of the simulation domain in dimensionless units along the $x$ and $y$ directions is $30$, and the deformation is subject to a vanishing boundary conditions on the lines $y,z=\pm15$. The spacing of the numerical grid is 0.3 and the number of effective digits of precision is three. The dimensionless number $\mathrm{Ma}\cdot\chi\cdot\tau_{l}/(2\tau_{th})$ is taken as unity. The intensity, $I$, is taken as $I(r)=exp(-(r-r_{0})^{2})$, where $r_{0}=0$ corresponds to maximal temperature and thinning at the origin, whereas $r_{0}=5$ corresponds to maximal temperature and thinning on a circle of radius $r_{0}$, which leads to a drop-like structure.

## 7 Comparison of sensitivities to thickness changes due to Fresnel reflection and coupling into SPP

Consider the value of the ratio $s$, defined as $s\equiv\Delta R/\Delta w$ where $\Delta R$ is the change of reflectivity due to the corresponding change of TLD film thickness $\Delta w$. For Fabry-Perrot (FP) and SPP modes, the maximal value of the ratio $s$ , is given by $s_{max}^{FP}=\Delta R_{max}^{FP}/(\lambda/4n_{l})$ and $s_{max}^{SPP}=\Delta R_{max}^{SPP}/\Delta w(\Delta\theta_{1/2})$, respectively. Here, $\lambda/4n_{l}$ is the change of the dielectric thickness that corresponds to a maximal shift of reflectivity given by $\Delta R_{max}^{FP}=4r^{2}/(1+r)^{2}$ where $r=|(n_{g}-n_{l})/(n_{g}+n_{l})|$ is the Fresnel normal reflection coefficient; $\Delta w(\Delta\theta_{1/2})$ is the change of thickness which corresponds to twice the width of the angular half-width depth, $\Delta\theta_{1/2}$, of the corresponding coupling angle-thickness curve. Assuming $\Delta R_{max}^{SPP}=1$, and furthermore utilizing the values $n_{g}=1$, $n_{l}=1.39$, $\lambda=785$ nm, $\Delta\theta_{1/2}=4^{o}$ and the corresponding $\Delta w(\Delta\theta_{1/2})=15$ nm, yields enhancement of the proposed method by $s_{max}^{SPP}/s_{max}^{FP}\simeq15$.

## 8 Shape of thin liquid film under spinning on a substrate with periodic topography

Following [6], the corresponding solution of the governing equation (Eq.(14) in the main text) of TLD film under capillary and centrifugal forces in the quasi-static limit is given by the linear superposition of the functions $\varphi_{1,2,3,4}(X)$

$\begin{matrix} H_{t} & =1+\frac{q/(w-q)}{1+\Omega^{2}}+a_{t}\varphi_{1}(X)+b_{t}\varphi_{2}(X)+c_{t}\varphi_{3}(X)+\varphi_{4}(X) \\ H_{r} & =1+a_{r}\varphi_{1}(X)+b_{r}\varphi_{2}(X)+c_{r}\varphi_{3}(X) \end{matrix}$ (12)

Here, $t,r$ denote regions above the grating trench and ridge, respectively, and the corresponding functions are given by

$\begin{matrix} \varphi_{1}(X) & =e^{-\lambda_{r,t}X} \\ \varphi_{2}(X) & =e^{\lambda_{r,t}X/2}\cos\left( \sqrt{3}\lambda_{r,t}X/2 \right) \\ \varphi_{3}(X) & =e^{\lambda_{r,t}X/2}\sin\left( \sqrt{3}\lambda_{r,t}X/2 \right) \\ \varphi_{4}(X) & =\frac{1}{3}[\left( 1+\frac{q/(w-q)}{1+\Omega^{2}} \right)-\left( 1+\frac{q/(w-q)}{1+\Omega^{2}} \right)^{4}] \end{matrix}$ (13)

where $\lambda_{r,t}$ take the following values in each one of the domains

$\lambda_{r}=(3\Omega^{2})^{1/3}; \lambda_{t}=\left( 3\Omega^{2}\left( \frac{q/(w-q)}{1+\Omega^{2}} \right)^{-4} \right)^{1/3}.$ (14)

The corresponding coefficients $a_{t,r}$, $b_{t,r}$, $c_{t,r}$ are then determined by the continuity of the fluid-air height as well as its first and second derivatives at points $X=-1/2$ and $X=(1+\Lambda)/2$.

Inserting the value $\Omega^{2}=2.06\cdot10^{-4}\ll1$ derived below Eq.(14) in the main text, we find that under surface tension and centrifugal forces the following values of mean thickness $w=36,60,90,150$ nm lead to the following values of $H=$ $5.9\cdot10^{-7}$, $3.6\cdot10^{-5}$, $1.2\cdot10^{-5}$, $3.9\cdot10^{-6}$ in units of $w-q$, i.e. much smaller than $1$ nm.

## 9 Numerical results of the effect of thin film’s undulation on the value of the resonant coupling angle





Figure 3: Numerical simulation results presenting the effect of the periodic undulation amplitude on the resonance coupling angle. (a) Presents the value of the resonant coupling angle as a function of the dimensionless undulation peak to peak height, $H/w$, and (b) presents the reflectance curve for TLD films of thickness $50$ nm with different peak to peak undulation with values $H=0,10,30$ nm, indicating that periodic and symmetric undulation does not introduce a significant shift to the resonant coupling angle.

Let us determine the effect of thin film undulation derived above, on the value of the resonant coupling angle. To this end, we perform numerical simulations of an incoming plane wave on a grating of period $\Lambda$ described in Fig. 4a in the main text. The corresponding shift in the resonant angle is described in Fig. S3a. Remarkably, the corresponding angle shift is a very weak function of $H/w$, even for values of $H$ comparable to $w$. Fig. S3b furtheremore indicates that, the reflectance curve is practically unaffected by the periodic symmetric undulation. Periodic undulations which are not symmetric relative to the grating are expected to modify the resonant coupling angle for sufficiently large amplitude, and these are not considered in this work.

## 10 Experimental setup, fabrication details, and sample preparation

Fig. S4 presents the schematic description of the optical setup we employed in our experiments. In particular, Fig. S4a and Fig. S4b describe the experimental setups we used for real-space and $k$-space measurements, respectively, whereas Fig. S4c shows the pattern projection setup. The low power near infra-red (NIR) laser diode ($\lambda=785$ nm) is used as a probing-beam, whereas the higher power argon laser ($\lambda=488,$ $514$) nm is used as a heating-beam; these two beams were brought to the same optical path by means of a short pass filter (SPF). Upon reflection from the sample, the light was imaged to CMOS camera by utilizing a 50:50 beam splitter (BS). Real-space imaging is achieved by placing the camera in the the back focal plane (BFP) of the tube lens, and by placing an additional lens L1 in the illumination path of the probing beam in order to deliver a plane wave to the sample; L1 is mounted on a translation stage which can change its position in a direction perpendicular to the optical axis. $k$-space imaging is achieved by inserting lens L3 after lens L2 in order to complete a 4f imaging system to image the BFP of the microscope objective, and by removing lens L1 in order to focus the probing beam onto the sample. The heating pattern projector unit, described in Fig. S4c, is comprised of a transparency mask and an additional 15 inch lens L4 placed before SPF. For droplet generation in white light imaging setup we used $25.4$ mm diameter Axicon (Thorlabs AR coated UC fused silica) with deflection angle of $0.23^{o}$ and with reflectence less than $1$ % in the range $350$ - $700$ nm, and heating beam which employs $400$ mW laser power source of wavelength $\lambda=532$ nm.


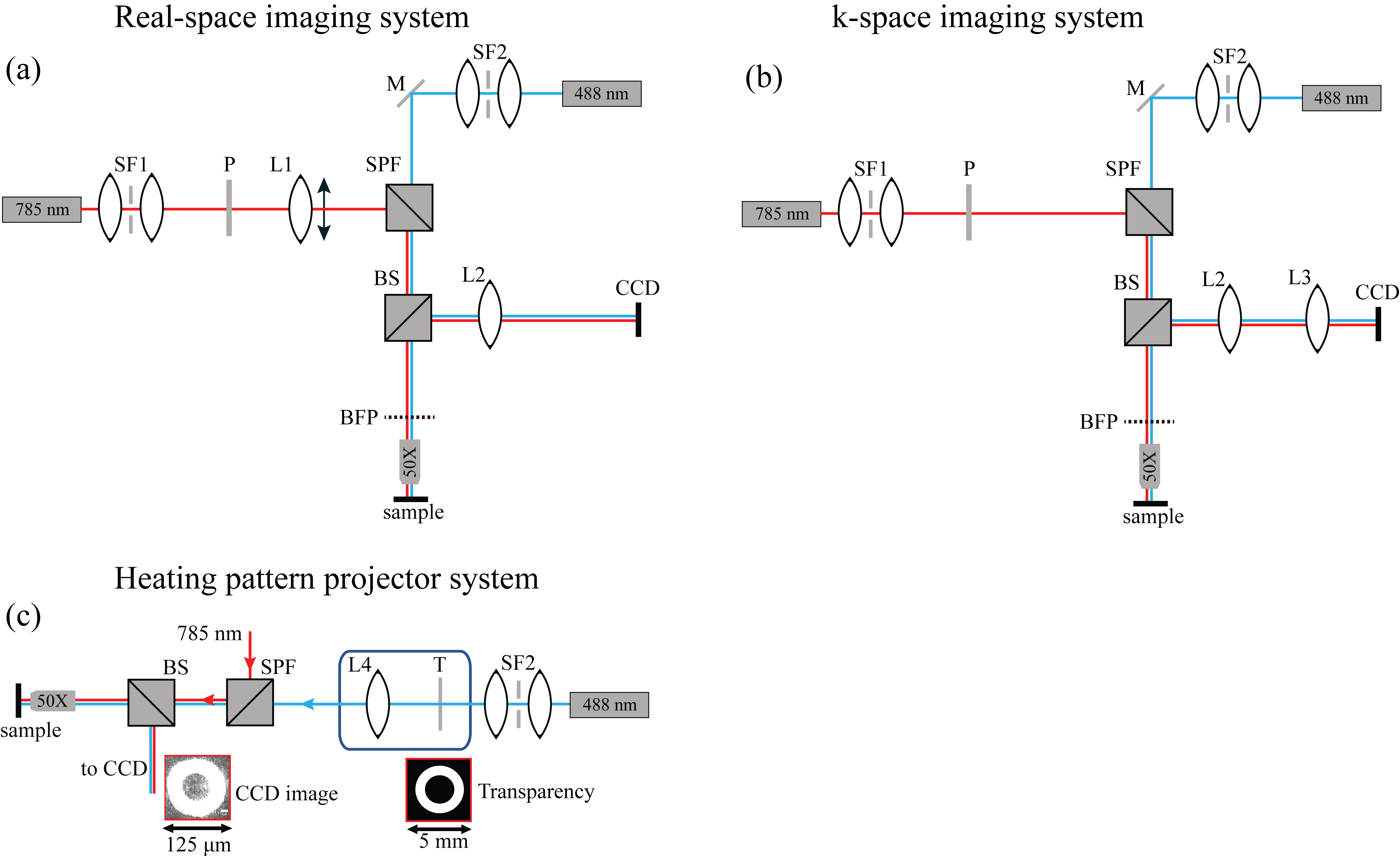


Figure 4: Experimental setup for: (a) real-space and (b) k-space imaging systems, and (c) the pattern projection setup to induce droplet directly from TLD film. SF1 and SF2 stand for spatial filters (SFs) comprised from a pair of lenses and a pinhole. Real-space imaging is achieved by placing the camera in the back focal plane (BFP) of the tube lens, and by placing an additional lens L1 in the illumination path of the probing beam in order to deliver a plane wave to the sample; L1 is mounted on a translation stage which can change its position in a direction perpendicular to the optical axis. k-space imaging is achieved by inserting lens L3 after lens L2 in order to complete a 4f imaging system to image the BFP of the microscope objective, and by removing lens L1 in order to focus the probing beam onto the sample. Other components: SPF - short pass filter, P - polarizer, L2 - tube lens, BS - 50:50 beam splitter. The heating pattern projector unit described in (c) is comprised of a transparency mask and additional 15 inch lens L4 placed before SPF.

For the metal substrate which supports SPP excitations and hosts TLD film we emloyed 600 nm gold grating of $50$% duty cycle, and $30$ nm depth grooves, which were fabricated by nanoimprint pattern transfer and lithography, as described in the followin. Using electron-beam evaporation, a silicon substrate was layered with $5$ nm of Ti as adhesion and $200$ nm of Au to form the grating bulk material. A nanoimprint resist bilayer was spun and soft-baked on top with $75$ nm of PMMA forming the underlayer and $160$ nm of upper layer resist (AR-UVP), onto which a polymeric mold containing the grating features was aligned and stamped while curing the imprinted resist (EVG aligner). After mold/sample separation, reactive ion etch (RIE) recipes were used to remove residual top and bottom layer resists within the pattern trenches. The pattern was transfered from imrpint resist to a metal mask by $30$ nm of Cr deposition and acetone liftoff. Using the Cr mask, an additional RIE recipe was used to directly remove $30$ nm of Au within the exposed trenches, followed by wet etch removal of Cr. The pattern depth, duty cycle, and periodicity were confirmed by AFM measurements (Nanonics).

To form a TLD film, silicone oil (Fisher Scientific) of refractive index $1.39$ was spun onto the grating by repeated intervals of spin coating at $10,000$ rpm. Baseline thickness of the prepared fluid film was measured by spectral reflectance to be on average $176$ nm by a separate optical profilometer (Filmetrics F20). A complete spin curve for $3-12$ each spin being $1.5$ min in duration, was obtained to prepare baseline average fluid thickness from $175$ to $700$ nm.

## 11 Movie demonstrating $\boldsymbol{k}$-space thickness measurement of TLD film

Experimental $k$-space imaging movie, demonstrating dynamical nanometric thickness change of a thin silicone oil film as a function of time due to optically-driven TC effect. Early film thinning is triggered by a local decrease of surface tension values, which is then followed by a healing dynamics after the heating beam is switched off. Top, presents the resonant angles along the central line (i.e. $k_{y}=0$) of the $k$-space diagram, whereas bottom presents the corresponding thickness at the illuminated diffraction-size region.

**References**

1 Oron, A. et al. Long-scale evolution of thin liquid films. *Reviews of Modern Physics* **69**, 931-980 (1997).

2 Levich, V.G., Physicochemical Hydrodynamics (Prentice Hall, 1962).

3 Rubin, S. & Fainman, Y. Nonlocal and nonlinear surface plasmon polaritons and optical spatial solitons induced by the thermocapillary effect. *Physical Review Letters* **120**, 243904 (2018).

4 Stillwagon, L. E. & Larson, R. G. Leveling of thin films over uneven substrates during spin coating. *Physics of Fluids A*: *Fluid Dynamics* **2**, 1937-1944 (1990).

5 Johnson, P. B. & Christy, R. W. Optical constants of the noble metals. *Physical Review B* **6**, 4370 (1972).

6 Wu, P. Y. & Chou, F. Complete analytical solutions of film planarization during spin coating. *Journal of The Electrochemical* *Society* **146.10**, 3819-3826 (1999).
